# rchime: an R package for detecting chimeras in amplicon sequencing data

**DOI:** 10.1128/mra.01048-26

**Published:** 2026-10-21

**Authors:** Sarah L. Westcott, Patrick D. Schloss

**Affiliations:** 1 Department of Microbiology & Immunology, University of Michigan 1259, Ann Arbor, Michigan, USA; Indiana University Bloomington, Bloomington, Indiana, USA

**Keywords:** microbial ecology, 16S rRNA, chimera, microbiome

## Abstract

A longstanding challenge in analyzing amplicon sequencing data is the presence of chimeras, which are PCR products that are formed by two or more templates. rchime is an R package that implements the UCHIME algorithm, which efficiently screens amplicon sequence collections for chimeras.

## ANNOUNCEMENT

A persistent problem in analyzing 16S rRNA gene sequence data and other amplicon sequencing data is the detection and removal of sequences from PCR products by two or more templates (1–4). Over the past 30 years, several methods have been developed for detecting these chimeric sequences (5–7). The most effective approach is the algorithm implemented in UCHIME and USEARCH (7, 8). This approach can be implemented using a reference-based approach using an external database of trusted sequences or in a *de novo* approach by placing greater trust in more abundant non-chimeric sequences in a data set.

Aside from the original UCHIME and USEARCH tools, a faster version of the algorithm has been available for free through the open-source VSEARCH software package (9). These tools are accessible through the mothur and qiime2 software packages (10–12). We created the rchime package to make the popular algorithm available within the R programming environment, which an increasing number of researchers are using for their data analysis and visualization.

Users can detect chimeras using either the *de novo* or reference-based approaches. In either approach, users provide an R data frame including DNA sequences and names of the sequences. For the *de novo* approach, abundance data are provided for each sequence in each sample it is found. In the reference-based approach, an additional data frame of reference sequences and names are provided. A built-in data frame is provided within the package that contains the “gold” database of trusted non-chimeric sequences (6). Either approach returns a list indicating which sequences are chimeric and diagnostic data indicating the nature of the chimeras. To accelerate chimera detection. the code has been parallelized.

The rchime package is built using the Rcpp to integrate our own and modified versions of existing C++ code from VSEARCH into R. The package can be used on any operating system. The RcppThread package was also used to facilitate parallelization of the code. In addition to supplying the sequence and abundance data as a data frame, it can also be provided as a strollur object created using the strollur package (13).

To illustrate the similar performance of VSEARCH and rchime, we used the example data included with rchime that contains 6,084 unique 16S rRNA gene sequences from 20 samples on a MacBook Pro. Because VSEARCH does not separate sequences by sample, we first treated the sequences as coming from the same sample. The *de novo* approach required 2.6 s in VSEARCH, and rchime required 2.7 s (median of 50 iterations). Next, we detected chimeras in each sample separately with the *de novo* approach using the VSEARCH implementation in mothur, which took 5.0 s, and rchime, which took 4.2 s. The sequences classified as being chimeras were the same across implementations.

By integrating commonly used tools like rchime, clustur (14), and phylotypr (15) into the R programming environment, we hope that researchers will experience easier installation, a more gentle learning curve, and better integration of tools than they often experience using stand-alone tools with a command line interface.

## Data Availability

rchime (currently version 0.1.2) can be accessed through the Comprehensive R Archive Network (CRAN; DOI: https://doi.org/10.32614/CRAN.package.rchime), and developmental versions are available through the project’s GitHub website (https://github.com/mothur/rchime). The package’s GitHub repository includes the package’s source code, the website’s source code, automated tests written using the testthat R package, and an issue tracker where users can post questions, bug reports, and suggestions for future features. A pkgdown version of the documentation, including multiple articles describing its use, is hosted at https://mothur.org/rchime. The rchime package is available under the GPL License (v3).
